# The decrease of consistence probability: at the crossroad of catastrophic transition of a biological system

**DOI:** 10.1186/s12918-016-0295-y

**Published:** 2016-08-01

**Authors:** Pei Chen, Yongjun Li

**Affiliations:** School of Computer Science and Engineering, Wushan Road, 510640 Guangzhou, China

**Keywords:** Dynamical network biomarker, Hidden Markov process, Pre-disease states

## Abstract

**Background:**

Unlike traditional detection of a disease state in which there are clear phenomena, it is usually a challenge to identify the pre-disease state during the progression of a complex disease just before the serious deterioration, not only because of the high complexity of the biological system, but there may be few clues and apparent changes appearing until the catastrophic critical transition occurs.

**Results:**

In this work, by exploiting the different dynamical features between the normal and pre-disease states, we present a hidden-Markov-model (HMM) based computational method to identify the pre-disease state and elucidate the essential mechanisms during the critical transition at the network level. Specifically, by considering the network variation and regarding that the pre-disease state is the end or shift-point of a stationary Markov process, a consistence score is proposed to measure the probability that a system is in consistency with the normal state. As validation, this approach is applied to detect the upcoming critical transition of complex systems based on both the dataset generated from a simulated network and the rich information provided by high-throughput microarray data. The effectiveness of our method has been demonstrated by the identification of the pre-disease states for two real datasets including HCV-induced hepatocellular carcinoma and virus-induced influenza infection.

**Conclusion:**

From dynamical view point, the critical-transition phenomena in many biological processes are of some generic properties, which can be detected by the established method.

## Background

Recently, evidence suggests that the deterioration of many complex diseases is not necessarily smooth but abrupt, that is, the sudden change of system state exists widely during the progression of complex diseases. For example, some chronic diseases such as cancer, the malignant deterioration may arise within a period of short-time progression, while before such catastrophic transitions the disease such as chronic inflammation may progress gradually for years of long incubative duration [1–5]. In other words, during the progression of illness there is a sudden critical state transition from a relatively healthy stage to a seriously diseased stage. For many complex diseases, it is crucial to detect such critical state transition in advance so as to prevent or at least get ready for such a catastrophic event. However, it is still a challenge work to signal the upcoming critical deterioration since the state of the system may show little apparent change before the tipping point is really reached. This is also the reason why diagnosis based on traditional biomarkers may fail to indicate a pre-disease state. A possible approach to study the warning signal of the sudden deterioration is to explore and analyze the dynamical features generated from the early abnormalities in distinct time-series prior to the emergence of the apparent malignancy. Therefore, in order to describe the underlying dynamical mechanism of complex diseases, their evolutions are often modeled as time-dependent nonlinear dynamical systems, in which the abrupt deterioration or qualitative transition is viewed as the state transition or phase shift at a bifurcation point [6]. We particularly focus on the complex diseases with sudden deterioration phases or critical transition points during their progressions.

**Fig. 1:**
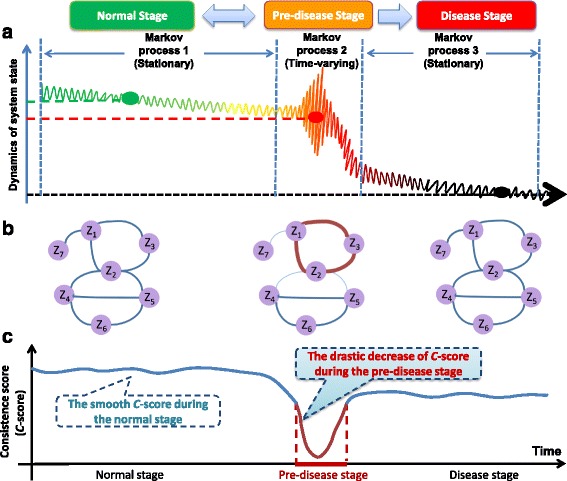
Outline for identifying the pre-disease state by using hidden Markov model. **a** The progression of a complex disease can be generally divided into three states, i.e., the normal state, the pre-disease state, and the disease state. Both the normal and disease states are stable with high resilience, while the pre-disease state, a critical stage, is unstable with low resilience and sensitive to the parameter changes. Thus the biological progression of diseases in both the normal and disease states are modelled as stationary Markov processes, and that in the pre-disease state is described by a time-varying Markov process. The detection of the onset of a pre-disease state is equivalent to the identification of the end point of the stationary Markov process in a normal state. **b** The three networks stand for the evolution of the system respectively in three states. The thickness of links stands for the correlation between each pair of nodes. It can be seen that when the system is in the pre-disease state, a few nodes form a special subnetwork among which the correlations abruptly increase, while the correlations between the subnetwork and other nodes decrease. It is worth noting that such critical phenomenon appears only in the pre-disease state. **c** On the basis of hidden Markov model (HMM), we propose a consistence score (*C*-score) to measure the dynamical change of system, that is, the *C*-score curve is expected to be smooth when the system is in a stationary Markov process, while the *C*-score drastically decrease when the system is in a time-varying Markov process. Thus, it is possible to detect the imminent critical transition by identifying the sudden change of the *C*-score

It was previously hypothesized that the disease progression can be modeled into three states (Fig. 1a): (A) a normal state (or a before-transition stage), representing a relatively healthy stage with high stability to external perturbations; (B) a pre-disease state (or a pre-transition stage), defined as the prelude to catastrophic deterioration into the disease state, occurring before the imminent phase transition point is reached, therefore, with low stability due to its dynamical structure; (C) a disease state (or an after-transition stage), representing a seriously deteriorated stage possibly with high stability, because the system usually finds it difficult to recover or return to the normal state even after treatment [7–9]. This is supported by the observations that there is usually sudden health catastrophic shift during the gradual progression of many chronic diseases [10–13]. Recently, a concept called dynamical network biomarker (DNB) was presented to detect the impending critical transition, or equivalently, the pre-disease state [14, 15]. The DNB method and its subsequent modifications have been successfully applied to real biological and clinical data, and identified the early-warning signals of the sudden deterioration of several complex diseases [16–21].

In this work, by exploring the distinct dynamical features between the correlation networks respectively generated in normal and pre-disease state, we developed a computational method on the basis of the hidden Markov model (HMM) for identifying the pre-disease state before the critical point is really reached during the biological process of complex diseases. Specifically, it is natural to model the progression of a biological system in a normal state as a stationary Markov process, since the normal state is a stable state and with high resilience. The pre-disease state is modelled as the time-varying Markov process due to its unstable nature and high sensitivity to even small perturbation. The disease state is another stationary Markov process in view of its high stability (see Fig. 1a). Identifying the pre-disease state is then equivalent to detecting the end of the stationary Markov process. Utilizing the time-course data, we presented the computational method and algorithm on estimating the possibility of supposed termination of Markov process at each candidate sampling point. Specifically, by exploring the critical phenomena of network structure in dynamics (Fig. 1b), a consistence score (*C*-score) was proposed to signal the upcoming critical transition, i.e., the drastic decrease of *C*-score implies the onset of a pre-disease state, in contrast to the relatively smooth *C*-score in either a normal or disease state (Fig. 1c). To demonstrate the effectiveness of our method, we applied the algorithm to a simulated regulation network and two sets of real data, the microarray dataset of HCV-induced dysplasia and hepatocellular carcinoma (HCC) (GSE6764) and live influenza infection (humans) caused by H3N2 virus (GSE30550). The pre-disease states were successfully identified for both numerical simulation and real datasets, and thus signaling the imminent critical transitions.

## Methods

We first present the theoretical basis, i.e., the dynamical properties of a complex system near the tipping point, and then illustrate the preprocessing of real datasets and the detail algorithm.

### Theoretical basis

Disease progression or its biological process can be generally divided into three states or stages, i.e., (A) the normal stage, (B) the pre-disease stage, and (C) the disease stage (Fig. 1a). The normal stage is a stable state with high resilience and robustness stage, during which the state may change slowly and thus is modelled as a stationary Markov process. The pre-disease stage is unstable and defined as the limit of the normal stage just before the occurrence of catastrophic phase shift. It is sensitive to perturbation including noise or external interference that leads to the change of system parameters, thus still reversible to the normal stage given appropriate interventions. Therefore, the system progression during a pre-disease stage is considered as a time-varying Markov process, during which the state-transition probability may fluctuate from time to time. However, further progression of the illness led by persistent effects of perturbation may trigger a drastic state change into the disease stage, the other stable state described as the second stationary Markov process, which is usually difficult to return to the normal state even with intensive interventions. Hence, it is crucial to detect the pre-disease state so as to prevent qualitative deterioration into an irreversible stage. On the basis of the above settings, detecting the imminent critical transition is equivalent to identifying the end point (or switching point) of the stationary Markov process (Fig. 1). Besides, we investigate the different dynamical features between the correlation network respectively generated from normal and pre-disease state, i.e., comparing the differential links from adjacent time points.

Based on such study design, we carry out theoretical derivation in the following sections.

### Markov process of the network evolution near the critical point

We describe the theoretical derivation of our computational method, and introduce the qualitative behaviors in dynamics of biological variables to characterize the critical transition. The dynamics for the progression of complex diseases is very complicated either before or after the critical transition, and therefore the state equations are generally constructed in a high-dimensional space with a large number of variables and parameters. Therefore, it is a difficult task to construct an accurate mathematical model describing the dynamical behavior of the system during the biological process. Thus we aim at developing a model-free method to detect the critical signal.

We consider a discrete-time dynamical system in generic form

1 $$ Z(k+1)=f(Z(k); P).   $$

where *Z*(*k*)=(*z*_1_(*k*),…,*z*_*n*_(*k*)) is an *n*-dimensional state vector or variables at time instant *k* that represents gene or protein expressions, while *P*=(*p*_1_,…,*p*_*s*_) is a parameter vector or driving factors that represent slowly changing factors, e.g., genetic factors (SNP, CNV, etc.) and epigenetic factors (methylation, acetylation, etc.). *f*:**R**^*n*^×**R**^*s*^→**R**^*n*^ are generally nonlinear functions. Furthermore, the following conditions are assumed to be held for system (1). **(1)**$\bar {Z}$ is a fixed point of system (1) such that $\bar {Z}= f(\bar {Z}; P)$. **(2)** There is a value *P*_*c*_ such that one or a pair of eigenvalues of the Jacobian matrix $\left.\frac {\partial f(Z; P_{c})}{\partial Z}\right |_{Z=\bar {Z}}$ is equal to 1 in the modulus. **(3)** When *P*≠*P*_*c*_, the eigenvalues of (1) are not always equal to 1 in the modulus. These three conditions with other transversal conditions imply that the system undergoes a phase change at $\bar {Z}$ or a codimension-one bifurcation when *P* reaches the threshold *P*_*c*_.

For system (1) near $\bar {Z}$, before *P* reaches *P*_*c*_, the system is supposed to stay at a stable fixed point $\bar {Z}$ and therefore all the eigenvalues are within (0,1) in modulus. The parameter value *P*_*c*_ at which the state shift of the system occurs is called a bifurcation parameter value, or a critical transition value.

Now we consider the linearized approximate equations of Eq. (1). Specifically, by introducing new variables *Y*(*t*)=(*y*_1_(*t*),…,*y*_*n*_(*t*)) and a full-rank transformation matrix *S*=(*s*_*ij*_)_*n*×*n*_ satisfying *J*=*SΛS*^−1^, i.e.,

2 $$ Y(t)=S^{-1}(Z(t)-\bar{Z}).   $$

we have

3 $$ Y(t+1)=\Lambda Y(t)+ \zeta(t).   $$

where *ζ*=(*ζ*_1_,…,*ζ*_*n*_) are small Gaussian noise with zero means. *ζ*_*i*_ has a small standard deviation *σ*_*i*_ for all *i*, and covariances *κ*_*ij*_=Cov(*ζ*_*i*_,*ζ*_*j*_).

Without loss of generality, the diagonalized matrix *Λ*=(*λ*_1_,…,*λ*_*n*_) is assumed to have each *λ*_*i*_ between 0 and 1. Among the eigenvalues of *Λ*, the largest one (in modulus), say *λ*_1_, first approaches to 1 in modulus when parameter transition *P*→*P*_*c*_ occurs. The eigenvalue *λ*_1_ characterizes the system’s rate of change around the fixed point and is called the dominant eigenvalue. The normal state corresponds to the period with |*λ*_1_|<1, whereas the pre-disease stage corresponds to the period with |*λ*_1_|→1. Without the loss of generality, the first variable *y*_1_ in *Y* is assumed to be associated with *λ*_1_. Calculating the statistical indices, it is clear that the Pearson’s correlation coefficient (PCC) is of the following expression

$${} {{\begin{aligned} &\ \ \ \ \ \text{PCC}(z_{i}, z_{j})= \frac{\text{Cov}(z_{i}, z_{j})}{\sqrt{\text{Var}(z_{i})\text{Var}(z_{j})}}\\ &=\frac{s_{i1}s_{j1}\frac{\kappa_{11}}{1-{\lambda_{1}^{2}}} +\sum\limits_{k=2}^{n}s_{ik}s_{jk}\frac{\kappa_{kk}}{1-{\lambda_{k}^{2}}} +\sum\limits_{\substack{k, m=1\\ k\neq m}}^{n}s_{ik}s_{jm}\frac{\kappa_{km}}{1-\lambda_{k} \lambda_{m}}} {\sqrt{\left(\sum\limits_{k=1}^{n}\frac{s_{ik}^{2}\kappa_{kk}}{1-{\lambda_{k}^{2}}} \,+\,\sum\limits_{\substack{k, m=1\\ k\neq m}}^{n}\frac{s_{ik}s_{im}\kappa_{km}}{1-\lambda_{k} \lambda_{m}}\right) \left(\sum\limits_{k=1}^{n}\frac{s_{jk}^{2}\kappa_{kk}}{1-{\lambda_{k}^{2}}} \,+\,\sum\limits_{\substack{k, m=1\\ k\neq m}}^{n}\frac{s_{jk}s_{jm}\kappa_{km}}{1-\lambda_{k} \lambda_{m}}\right)}}. \end{aligned}}} $$

Obviously, there are three cases as follows.

- when *s*_*i*1_≠0 and *s*_*j*1_≠0, $\lim \limits _{|\lambda _{1}|\rightarrow 1}\text {PCC}(z_{i}, z_{j})\rightarrow 1$;
- when *s*_*i*1_≠0 and *s*_*j*1_=0, $\lim \limits _{|\lambda _{1}|\rightarrow 1}\text {PCC}(z_{i}, z_{j})\rightarrow 0$;
- when *s*_*i*1_=0 and *s*_*j*1_=0, $\lim \limits _{|\lambda _{1}|\rightarrow 1}\text {PCC}(z_{i}, z_{j})\rightarrow P_{ij}$, where *P*_*ij*_ is a bounded value.

Hence, close to a tipping point, among the original variables *Z*=(*z*_1_,...,*z*_*n*_) there is a dominant group which is composed of dominant variables *z*_*i*_=*s*_*i*1_*y*_1_(*k*)+⋯+*s*_*in*_*y*_*n*_(*k*) with *s*_*i*1_≠0. It is clear from the above derivation that the correlation between a pair of dominant variables increases sharply as the dominant eigenvalue |*λ*_1_|→1, while the correlation between a dominant variable and any other molecule decreases sharply. It also should be noted that such critical change of the correlation only appears when the system approaches to the critical tipping point, or equivalently, the system is in a pre-disease state. By employing this dynamical feature between a normal state (when the system is far from the tipping point) and a pre-disease state (when the system is in the vicinity of the tipping point), it is possible to detect the early-warning signal of the critical transition based on the hidden Markov model.

### Identifying the end of Markov process and the algorithm of HMM-based method

Based on the dynamical characteristics of a complex biological system and the discussion above, it is natural to regard the critical transition as the switch from a stationary Markov process (i.e., the normal state) to a time-varying Markov process (i.e., the pre-disease state). Therefore, identifying the pre-disease state is equivalent to detecting the end point or switch point of a stationary Markov process. To present the computational method, we first introduce the following symbols.

- Denote the stationary Markov process as *M*_1_, and the time-varying Markov process as *M*_2_.
- Denote the time variable as *t*, and the progression of the system along time series as *t*∈{1,2,...,*T*−1,*T*,...}.
- Denote the observed sequence up to time point *t* as *O*={*o*_1_,*o*_2_,...,*o*_*t*−1_,*o*_*t*_}, where *o*_*t*_ represents the sample set derived at time point *t*.
- Denote the state sequence up to time point *T* as {*s*_1_,*s*_2_,...,*s*_*T*−1_,*s*_*T*_}, i.e., the state of the system is *s*_*T*_ at time point *t*=*T*, or equivalently, *s*_*T*_=*State*(*o*_*T*_).

Specifically, it is assumed that a biological system is initially in the normal state, or equivalently, the progression of the system is in a stationary Markov process *M*_1_. Then for the progression of the system along a time series {1,2,...,*T*−1,*T*,...}, we propose a consistence score (*C*-score) to measure the probability of the system being in the same stationary Markov process, i.e.,

4 $${} C(T)\,=\,P_{T}(s_{T}\,=\,M_{1}|\,s_{1}\!\,=\,M_{1}, s_{2}\,=\,M_{1},..., s_{t\,-\,1}\!\,=\,M_{1},\, \theta_{t-1}, O).   $$

For each candidate time point *t*=*T*, the high value of *C*-score presents that the progression of the system at *t*=*T* is consistent with the stationary Markov process *M*_1_, i.e., it is still in the stationary Markov process, while the sudden decrease of *C*-score illustrates the low consistence with *M*_1_ (Fig. 1c), and the progression of the system is no longer in the stationary Markov process. Therefore, the abrupt change of *C*-score identifies the pre-disease state and indicates the upcoming critical transition.

From the third time point or sampling stage during a time series, we regard each point/stage as a candidate transition point/stage. In order to validate whether a candidate time point *t*=*T* (*T*=3,4...) is the changing or switching point from the stationary Markov process to the time-varying Markov process, we carry out an iterative process as the following two steps.

1. Train a hidden Markov model (HMM) *θ*_*T*−1_=(*A*,*B*,*π*) on the basis of an observed sequence {*o*_1_,*o*_2_,...,*o*_*T*−1_}, i.e., the preceding *T*−1 sets of samples generated from time points 1,2,...,*T*−1. The stationary Markov process in the normal state is actually described by the trained HMM.
2. Calculating the *C*-score based on the observation {*o*_*T*_} and the trained HMM *θ*_*T*−1_. If there is a drastic decrease of *C*-score, then the iterative process end up with *t*=*T* being the switching point, at which the biological system is in the pre-disease stage. Otherwise go to back to the training step for next time point *t*=*T*+1.

First, to train an HMM *θ*_*T*−1_=(*A*,*B*,*π*) where the subscript *T*−1 of *θ* represents that the HMM is derived from the training samples up to time point *t*=*T*−1, we need to estimate a state transition matrix *A*, an emission matrix *B*, and a probability vector for the initial state *π*. For a network with *n* nodes and *m* links where each node represents a bio-molecule and each link represents the correlation between two nodes, suppose at a sampling time point *t*∈{1,2,...,*T*−1,*T*,...} there are *w* samples for each node *z*_*i*_, i.e., $\{{z_{i}^{1}} (T-1),{z_{i}^{2}} (T-1),...,{z_{i}^{w}} (T-1)\}$. Then through leaving-one-out procedure we obtain *w* Pearson’s correlation coefficients (PCCs) between any two nodes *z*_*i*_ and *z*_*j*_, i.e., {*PCC*_1_(*z*_*i*_,*z*_*j*_),*PCC*_2_(*z*_*i*_,*z*_*j*_),...,*PCC*_*w*_(*z*_*i*_,*z*_*j*_)} where each *PCC*_*k*_(*z*_*i*_,*z*_*j*_) (*k*=1,2,...,*w*) is calculated based on *w*−1 samples for *z*_*i*_ and *z*_*j*_. To train *A* and *B* based on an unsupervised learning procedure, we have the following steps.

**A. Estimate the distribution of each link at a former time point*(T−2)*** Under the assumption that each correlation coefficient follows Gaussian distribution, we obtain the estimation of the distribution for link *PCC*(*z*_*i*_,*z*_*j*_)_*T*−2_ between two nodes *z*_*i*_ and *z*_*j*_ at time point *T*−2, i.e., based on the *w* correlation coefficients, we estimate the mean *μ*_*k*_(*T*−2) and standard deviation *σ*_*k*_(*T*−2) for each link *PCC*(*z*_*i*_,*z*_*j*_)_*T*−2_. Then we have the distribution $N(\mu _{k} (T-2), {\sigma ^{2}_{k}} (T-2))$.

**B. Determine the consistence vector for each variable at *(T−1)*** At time point *t*=(*T*−1), we have *m* links for the network, i.e., *link*_*k*_(*T*−1)=*PCC*(*z*_*i*_,*z*_*j*_)_*T*−1_ between two nodes *z*_*i*_ and *z*_*j*_ with *k*=1,2,...,*m*, and for each link there are *w* samples through leave-one-out procedure, i.e., $link_{k}(T-1)=\{lin{k_{k}^{1}},lin{k_{k}^{2}},...,lin{k_{k}^{w}}\}$. Let an index ${L_{k}^{s}} (T-1)\in \{0,1\}$ describe whether a correlation $lin{k_{k}^{s}}$ is consistent comparing with its former distribution $N(\mu _{k} (T-2), {\sigma ^{2}_{k}} (T-2))$, that is, whether the appearance of link at time *T*−1 is with large probability in the distribution $N(\mu _{k} (T-2), {\sigma ^{2}_{k}} (T-2))$. For each correlation *link*_*k*_(*T*−1) at time point *T*−1, we have

5 $${} {{\begin{aligned} {L_{k}^{s}} (T\,-\,1)\! =\!\left\{ \begin{array}{l} 0,\ \text{if}\,\,\, \,link_{k}\!\in [\mu_{k} (T-2)-\sigma_{k} (T-2),\mu_{k} (T\,-\,2)+\sigma_{k} (T-2)]\\ 1,\ \text{if}\,\,\,\, link_{k}\!\in (-\infty, \mu_{k} (T\,-\,2)-\sigma_{k} (T\,-\,2))\cup(\mu_{k} (T\,-\,2)+\sigma_{k} (T-2), +\infty) \end{array} \right..  \end{aligned}}}  $$

Obviously, ${L_{k}^{s}} (T-1)=0$ represents that the correlation *link*_*k*_(*T*−1) is consistent with the former distribution $N(\mu _{k} (T-2), {\sigma ^{2}_{k}} (T-2))$, while *x*_*k*_(*T*−1)=1 represents that the correlation *link*_*k*_(*T*−1) is inconsistent with the former distribution $N(\mu _{k} (T-2), {\sigma ^{2}_{k}} (T-2))$. Thus, for each sample of correlation $(lin{k_{1}^{s}} (T-1),lin{k_{2}^{s}} (T-1),...,lin{k_{m}^{s}} (T-1))$, the vector $L^{s} (t-1)=({L_{1}^{s}} (T-1),..., {L_{m}^{s}} (T-1))$ is the consistence vector at time *T*−1.

Let *#*0(*T*−1) and *#*1(*T*−1) respectively denote the number of value 0 and that of value 1 in an consistence vector *L*^*s*^(*T*−1) at *T*−1. Obviously, *#*0(*T*−1) + *#*1(*T*−1)=*m*, where *m* is the number of links in the network, among which there are *#*0(*T*−1) variables consistent with the former distribution $N(\mu _{k} (T-2), {\sigma ^{2}_{k}} (T-2))$, while *#*1(*T*−1) variables inconsistent with the former distribution $N(\mu _{k} (T-2), {\sigma ^{2}_{k}} (T-2))$.

According to above settings, we actually transform the observed correlation sample set *o*_*T*−1_=(*link*_1_(*T*−1),*link*_2_(*T*−1),...,*link*_*m*_(*T*−1)) into the corresponding consistence vector *o*_*T*−1_=(*L*^1^(*T*−1),*L*^2^(*T*−1),...,*L*^*m*^(*T*−1)).

**C. Training the HMM at *T−1*** In this step, we need to identify the state transition matrix *A* and the emission matrix *B* at (*T*−1), that is, training the HMM *θ*_*T*−1_=(*A*(*T*−1),*B*(*T*−1),*π*) on the basis of an observed sequence {*o*_1_,*o*_2_,...,*o*_*T*−1_}.

There are two possible states *W*_0_ and *W*_1_ in time point *t*−1. Then, we calculate the possibilities for each possible state transition and thus obtain the state transition matrix *A*(*T*−1)=(*a*_*ij*_(*T*−1))_2×2_, where

6 $$ a_{ij} (T-1)=P\left(s_{T-1}=M_{i} \,|\, s_{T-2}=M_{j}\right),   $$

with *i*,*j*∈{1,2}.

Besides, for the emission matrix *B*(*T*−1)=(*b*_*jk*_(*T*−1))_2×(*m*+1)_ where *b*_*jk*_(*T*−1) is the probability of the *k*th possible observation under the assumption that the system state is *W*_*j*_ at time *t*−1, i.e.,

7 $$ b_{jk} (T-1)=P\left(\#1(T-1)=k \,|\, s_{T-1}=M_{j}\right),   $$

where *j*∈{1,2} and *k*∈{0,1,2,...,*m*}. Obviously, there are *m*+1 possible observable cases for any correlation sample at *t*−1, i.e., case *#*1(*T*−1)=*k* with *k*∈{0,1,2,...,*m*}. In the case of an *m*-link biological network, case *#*1(*T*−1)=*k* reflects that there are *k* links differentially expressed in one observation (i.e., one sample) at *T*−1 comparing with their former expressions.

The initial state distribution *π*={*π*_1_,*π*_2_} is defined at time *T*−2, where

8 $$ \pi_{i} =P(s_{T-2}=M_{i}),   $$

with *i*∈{1,2}.

According to Baum-Welch algorithm, we build *A*, *B*, and *π* based on the training set {*o*_1_,*o*_2_,...,*o*_*T*−1_}, i.e., sample sets up to time *T*−1. The training process at time *T*−1 includes the following three steps.

- **Initialization** For *h*=0, set initial values for $a_{ij}^{0}$, $\,b_{jk}^{0}$, and ${\pi _{i}^{0}}$, we have the HMM *θ*^0^=(*A*^0^,*B*^0^,*π*^0^).
- **Update** For *h*=1,2,..., we have the update for $a_{ij}^{h}$, $\,b_{jk}^{h}$, and ${\pi _{i}^{h}}$ by recursion
  9 $${} a_{ij}^{h} =\frac{\sum\limits_{t=1}^{T-1}\xi_{t}(i,j)}{\sum\limits_{t=1}^{T-1}\gamma_{t}(i)}, \quad \!\!\!b_{jk}^{h} =\frac{\sum\limits_{t=1, \#1(T-1)=k}^{T-1}\gamma_{t}(k)}{\sum\limits_{t=1}^{T-1}\gamma_{t}(k)}, \quad{\pi_{i}^{h}} =\gamma_{1}(i),  $$
  where
  10 $$ \gamma_{t}(i)=P(s_{t}=M_{i}\,|\,O,\, \theta_{p})=\frac{P\left(s_{t}=M_{i},\, O\,|\,\theta_{p}\right)}{P(O\,|\,\theta_{p})}   $$
  and
  11 $$ {} {{\begin{aligned} \xi_{t}(i,j)=P\left(s_{t-1}=M_{i}, s_{t}=M_{j}\,|\,O,\, \theta_{p}\right)=\frac{P\left(s_{t-1}=M_{i}, s_{t}=M_{j},\, O\,|\,\theta_{p}\right)}{P\left(O\,|\,\theta_{p}\right)}  \end{aligned}}}  $$
  with *i*,*j*∈{0,1}. For *γ*_*t*_(*i*) and *ξ*_*t*_(*i*,*j*), the HMM *θ*_*p*_ used in the prior knowledge is that updated from the preceding step. For example, at the first iterative step, the HMM *θ*_*p*_ is *θ*^0^=(*A*^0^,*B*^0^,*π*^0^) based on the initial values. The observation sequence used in the prior knowledge is *O*={*o*_1_,*o*_2_,...,*o*_*T*−1_}.
- **Ending** When *h*=*H*, i.e., the *H*th-updating step, the recursion is terminated. Then
  12 $$ {\theta_{i}^{H}} =\left(A^{H},B^{H},\pi^{H}\right).   $$
  The HMM used in the testing process follows $\theta _{T-1}={\theta _{i}^{H}}$.

Under the assumption that the transition point is at *T*, or in other word, time point *T* is hypothesized as the end point of a stationary Markov process of the normal stage (see Fig. 1). Thus the onset of a pre-disease stage is the end of the stationary Markov process described as the trained HMM *θ*_*T*−1_. Therefore, at testing step in a candidate transition point *T*, we calculate the consistence score, i.e., *C*-score, based on the trained HMM *θ*_*T*−1_=(*A*(*T*−1),*B*(*T*−1),*π*).

According to the Markov chain, the *C*-score is

$${} \begin{aligned} &\ \ \ \ \ P_{T}(s_{T}=\!M_{1}\,|\, s_{T-1}\,=\,M_{1},...,s_{2}=M_{1}, s_{1}=M_{1},\theta_{T-1},\, O)\\ &=P_{T}(s_{T}=M_{1}\,|\, s_{T-1}=M_{1},\theta_{T-1},\, O)\\ &=\frac{P(s_{T-1}=M_{1}, s_{T}=M_{1}\,|\,\theta_{T-1},\, O)}{P(s_{T-1}=M_{1}\,|\,\theta_{T-1},\, O)}. \end{aligned} $$

The numerator

13 $${} {{ \begin{aligned} P(s_{T-1} =M_{1}, s_{T}=M_{1}\,|\,\theta_{T-1},\, O) =\frac{Q_{T-1}\!\left(s_{T-1}=M_{1}\right)a_{11}b_{1k}} {\sum\limits_{i=1}^{2}Q_{T-1}\left(s_{t-1}=M_{i}\right)a_{ij}b_{jk}},  \end{aligned}}}  $$

and the denominator

14 $$ P(s_{T-1} =M_{1}\,|\,\theta_{T-1},\, O) =\frac{Q_{T-1}(s_{T-1}=M_{1})} {\sum\limits_{j=1}^{2}Q_{T-1}(s_{T-1}=M_{j})},   $$

where *a*_11_ and *a*_*ij*_ is from the state transition matrix *A*=(*a*_*ij*_)_2×2_ in Eq. (6), *b*_1*k*_ and *b*_*jk*_ is from the emission matrix *B*=(*b*_*jk*_)_2×(*m*+1)_ in Eq. (7) while *k*=*#*1(*T*) represents that for the sample set *o*_*T*_ there are *k* variables with consistence index 1 in average, *Q* is the forward probability calculated based on standard forward algorithm. It should be noticed that in Eqs. (13) and (14) the backward probability is set to be 1, since samples *o*_*T*+1_,⋯ are not available when *T* is the testing time point.

According to above settings, given the HMM *θ*_*T*−1_, the calculation of HMM probability *P*_*T*_ at a candidate time point *T* only relies on the samples from *T*−1 and *T*. Obtaining the *C*-score *P*_*t*_ for every candidate time point, the time point *arg*_*t*_[*max*(*P*_*t*_)]_*t*=1,2,...,*T*_, is the transition point.

## Results

### Identifying the pre-transition state for a seven-node network

**Fig. 2:**
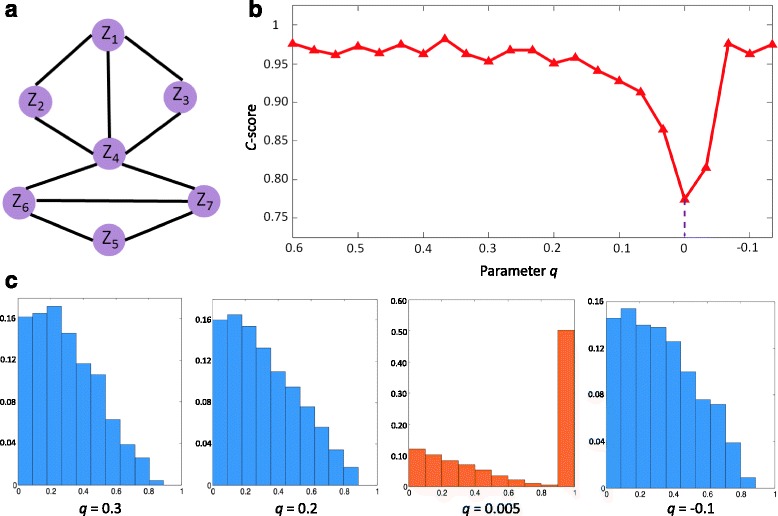
The validation of HMM-based method on a simulation dataset. To validate the sensitivity and effectiveness, our method was applied to the simulated dataset from a seven-node network. **a** The seven-node network, in which the nodes represent the biomolecules, and links represent the inter regulation between biomolecules. The network model described by a stochastic equation set is presented in **Result**. The tipping point is at a critical parameter value *q*_*c*_=0 in the theoretical model, where the system undergoes a critical transition. **b** From the *C*-score of the network, it can be seen that an abrupt decrease of the score signals the imminent critical transition at *q*_*c*_=0. **c** We illustrate the frequency distribution of the weight of links, i.e., the ratio of each emergent PCC value. It can be seen that when the system is in a normal state, i.e., the parameter *q* is far away from the critical value *q*_*c*_=0 (say, *q*=0.3,*q*=0.2), there are few edges with large PCC, which shows the weak correlation between the genes. However, while the parameter *q* approaches the critical value *q*_*c*_=0(*q*=0.005), the distribution is quite different, i.e., the ratio of 0.9-PCC-links increases considerably. The simulations were performed in MATLAB(R2013a) using the Euler-Maruyama integration method with the Ito calculus

To demonstrate the effectiveness of the computational method and the consistence score, we used a seven-node gene regulatory network (Fig. 2a) to show the detection of early-warning signals near a critical point. These types of gene regulatory networks are often used to study transcription, translation, diffusion, and translocation processes that affect gene regulatory activities [22]. The following seven differential equations represent the gene regulation of seven genes in a network where gene regulation is represented in a Michaelis-Menten form as the following Eq. (15), with the exception of the degradation rates, which are linearly proportional to the concentrations of the corresponding bio-molecules.

15 $$ {{} {\begin{aligned} \left\{\begin{array}{l} \frac{\mathrm{d}z_{1}(t)}{\mathrm{d}t}=\! \frac{(2- |q|)z_{2}(t)}{5(1+z_{2}(t))}-\frac{|q|+ 2}{5}\ z_{1}(t)+\zeta_{1}(t),\\ \frac{\mathrm{d}z_{2}(t)}{\mathrm{d}t} =\! \frac{(2-|q|)z_{1}(t) }{5(1+z_{1}(t))}-\frac{|q|+2}{5}\, z_{2}(t)+\zeta_{2}(t),\\ \frac{\mathrm{d}z_{3}(t)}{\mathrm{d}t} =\! \frac{2\,|q|-5}{5}+ \frac{5-2\, |q|}{10(1+z_{1}(t))}+\frac{5-2\,|q|}{10(1+z_{2}(t))}-z_{3}(t)+\zeta_{3}(t),\\ \frac{\mathrm{d}z_{4}(t)}{\mathrm{d}t} =\!\frac{2\,|q|-10}{5}\,+\,\frac{7-2\,|q|}{10(1+z_{1}(t))}\,+\,\frac{7-2\,|q|}{10(1+z_{2}(t))} \,+\,\frac{1}{5(1+z_{3}(t))}\,+\,\frac{2}{5(1+z_{6}(t))}\\ +\frac{2z_{7}(t)}{5(1+z_{7}(t))}-\frac{6}{5}z_{4}(t)+\zeta_{4}(t),\\ \frac{\mathrm{d}z_{5}(t)}{\mathrm{d}t} = -\frac{3}{10}+ \frac{3}{10(1+z_{6}(t))}+\frac{3z_{7}(t)}{10(1+z_{7}(t))}-\frac{7}{5}\,z_{5}(t)+\zeta_{5}(t),\\ \frac{\mathrm{d}z_{6}(t)}{\mathrm{d}t} =\frac{z_{7}(t)}{5(1+z_{7}(t))} -\frac{9}{5}\, z_{6}(t)+\zeta_{6}(t),\\ \frac{\mathrm{d}z_{7}(t)}{\mathrm{d}t} =\frac{z_{6}(t)}{5(1+z_{6}(t))} -\frac{9}{5}\, z_{7}(t)+\zeta_{7}(t), \end{array} \right.  \end{aligned}}}  $$

where *q* is a scalar control parameter and *ζ*_*i*_(*t*) (*i*=1,2,...,10) are Gaussian noises with zero means and covariances *κ*_*ij*_=Cov(*ζ*_*i*_,*ζ*_*j*_). *z*_*i*_ (*i*=1,...,10) represent the concentrations of mRNA-*i*. In Eq.(15), the degradation rates of mRNAs are $\left (\frac {2+|q|}{5}, \frac {2+|q|}{5}, 1, \frac {6}{5}, \frac {7}{5}, \frac {9}{5}, \frac {9}{5}\right)$. There is a stable equilibrium point $\bar {Z}=(\bar {z_{1}}, \bar {z_{2}},...,\bar {z_{10}})=(0,0, 0, 0, 0, 0, 0, 0, 0, 0)$. The differential equations Eq. (15) can be transformed into the difference equations *Z*(*k*+1)=*f*(*Z*(*k*),*P*) using Euler scheme with a small time interval 1. It is clear that there are seven distinct eigenvalues (0.67^|*q*|^,0.45,0.37,0.30,0.24,0.20,0.13) for the linearized system. Thus, the equilibrium point $\bar {Z}$ is stable when *q*∈(0,1]. There is a critical value *q*_*c*_=0. We aim to detect early warning signals that indicate the critical transition as a control parameter *q* approaches the critical value 0 from *q*>0. Applying the HMM-based approach to the system, we obtain the *C*-score curve as in Fig. 2b.

The numerical simulation shows that a drastic boost of the *C*-score, i.e., HMM probability, indicates the upcoming critical transition at parameter *q*=0 (Fig. 2b). To demonstrate the different dynamics of the system between the normal state and the pre-disease state, we illustrate the underlying frequency of links with different correlation values (Fig. 2c), from which it can be seen that there is a significant change in the frequency distribution of the links when the system is near a tipping point.

### Predicting critical transitions in real datasets

We applied the HMM-based method in three real experimental datasets, i.e., the microarray data for HCV-induced dysplasia and hepatocellular carcinoma (HCC) (GSE6764) and live influenza infection (humans) caused by H3N2 virus (GSE30550).

**Fig. 3:**
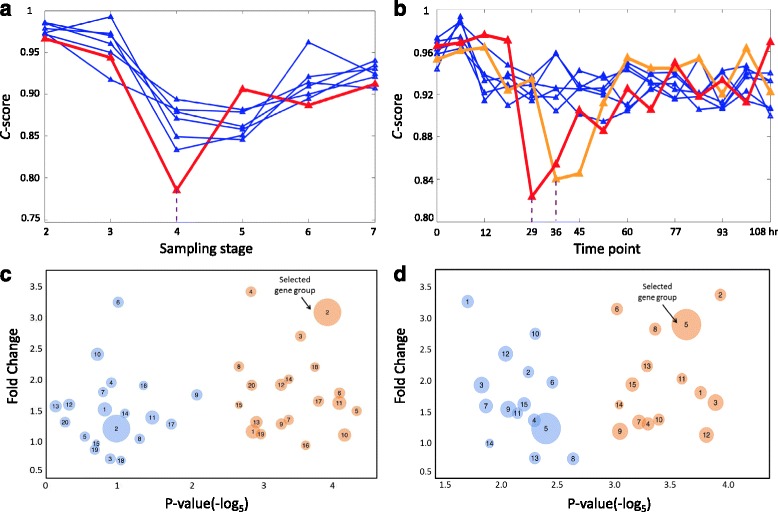
Detecting the critical transitions for two complex diseases. The HMM-based method was applied to identify the pre-disease states for two disease datasets. **a** The Consistence score based on the top differential-differential genes form each candidate transition time point for HCC. It can be seen that the most significant signal appears at the 4-th sampling stage (very early HCC). **b** The Consistence score based on the top differential-differential genes form each candidate transition time point for live influenza infection. It can be seen that the most significant signal appears at the 29 hours and 36 hours, which is in coincident with the clinical diagnosis. **c** We illustrate the clustering result respectively for genes at “very early HCC" stage (*orange bubbles*) and “low-grade dysplastic" stage (*blue ones*) for HCC. **d** We illustrate the clustering result respectively for live influenza infection at 29 h (*orange bubbles*) and 5 h (*blue* ones). Clearly, comparing with the control group, the *P*-value of each gene group is more significant at the critical stage than that at the early stage. The top differential-expression and fold-change gene groups were selected to proceed with functional analysis

We first present the application on HCV-induced HCC dataset, in which there are 7 sampling stages, i.e., cirrhosis, low-grade dysplastic stage, high-grade dysplastic stage, very early HCC stage, early HCC stage, advanced HCC stage and very advanced HCC stage. In these sampling stages, gene expression profiles of 75 tissue samples were analyzed representing the stepwise carcinogenic process from pre-neoplastic lesions (cirrhosis and dysplasia) to HCC, including four neoplastic stages (“very early HCC" to metastatic tumors). According to the presented method above, we regard that each sampling stage is a candidate transition point, i.e., the end point of a stationary Markov process in the normal state. To validate whether a candidate point is the transition one, there are the following four data-specific steps. First, to decrease the computational complexity, at each candidate point we selected top 5 % differential-expression genes through the rank of *P*-values from student t-test. Second, a network was constructed by mapping these selected genes to human protein-protein interaction (PPI) network from STRING (http://string-db.org/). Third, the normalized correlation values, i.e., PCCs, were calculated for the corresponding links at each stage. Fourth, at each candidate point, the *C*-score is then calculated (Fig. 3a). Clearly, there are seven probability curves respectively corresponding to seven groups of genes selected in distinct candidate points, i.e., each group of genes is differentially expressed at one time point. Among the probability curves in Fig. 3a, the red one presents the *C*-score calculated based on the network constructed at the “very early HCC" stage (the 4th sampling stage), from which it can be seen that at the 4th sampling stage the *C*-score shows the minimum probability which presents the least consistence of the system with the preceding state. This is in coincided with the previous result [9] and the observed biological phenotypes [23]. Thus the abrupt decrease of the *C*-score reflects the presence of a pre-disease state and indicates the imminent critical transition into a disease state (HCC stage). To further carry out functional analysis and elucidate the relation between top differential-expression genes and dysfunctional pathways, in Fig. 3c we also employed the clustering analysis through correlation at the identified pre-disease state (“very early HCC" stage), that is, we selected a clustering group of genes related to the differentially-expressed links (with *P*-value 1.91E-03 and around 3-fold change comparing with the control group) for further functional analysis.

**Fig. 4:**
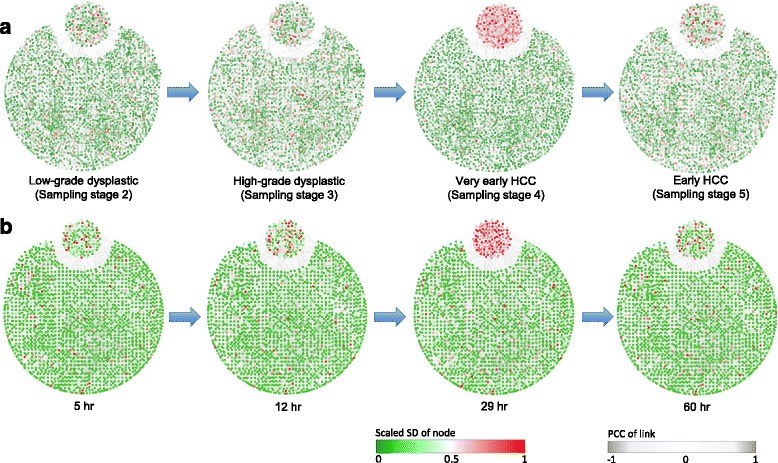
Dynamical changes in the network for the progression of two diseases. To validate the results from HMM-based method, we show the dynamical evolution of the network structure for the two diseases. For each network, the color of nodes represents the fluctuation of expression, and the thickness of links stands for the correlation between each pair of nodes. **a** For HCC, the figures show the dynamical changes of the human molecular network (3425 genes and 5826 edges) at 4 sampling stages, i.e., low-grade dysplastic stage, high-grade dysplastic stage, very-early HCC stage, early HCC stage. The subnetwork composed by selected 230 genes (from Fig. 3 c) is placed at the top corner. It can be seen that at the “very-early HCC" stage, there is a significant change in the selected subnetwork, which signals the upcoming deterioration into HCC. **b** For live influenza infection, it shows the dynamical changes of the human molecular network (3839 genes and 7281 edges) at 4 sampling time points, i.e., 5 hr, 12 hr, 29 hr, 60 hr. The subnetwork composed by selected 180 genes (from Fig. 3 d) is placed at the top corner. Obviously, at 29 hr the structure of the selected subnetwork exhibits the most significant change, which suggests the pre-disease stage around 29 hr and presents a warning signal for the imminent deterioration into the influenza infection. It is worth noting that the critical phenomena in the network structure only appear when the system is near the critical transition point

Figure 4a presents the dynamical evolution in the gene network based on the human molecular network with their functional interactions (protein-protein interactions and TF-target regulations). The selected gene group in Fig. 3c is placed at the top corner of each network. Clearly, at “very early HCC" stage the selected gene group are strongly correlated with wild fluctuation, which provides a significant signal from a network viewpoint and indicates the pre-disease state just before the deterioration into HCC, while other genes show no significant signal. Clearly, when the deterioration is impending, these selected genes form a special subnetwork, which actually guarantees the successful application of the HMM-based method, that is, this subnetwork exhibits the most significant changes in the links when the system is near a critical transition point. It can also be seen that, oppositely, neither the whole gene network nor the selected differential-expression genes present a signal before or after the transition, which shows the sensitivity of the *C*-score at the pre-disease state. In fact, the *C*-score reveals the existence of the pre-disease state, which, however, cannot be shown by any single bio-molecule. Therefore, the benefits brought by the HMM-based method in signaling the pre-disease state make the identification and management of high-risk cases more effective.

The functional analysis shows that some of the selected genes are highly relevant to the corresponding complex diseases, which validates the effectiveness of our method in a way. In the HCC study, many genes included in the top significant subnetwork relate to the response to HCV infection, especially the activation of the immune system and the dysfunctions associated with basic cell metabolism of hosts [23–25]. In the enrichment analysis, the most significant enriched pathways are related to the function of cell growth and cell metabolism, such as transcriptional misregulation in cancer, the Wnt signaling pathway and purine metabolism. The enriched pathways in cancer and hepatitis provide evidence that most of the genes related to differentially-expressed links may relate to the deterioration into HCC. These functional analysis implies the involvement of the differential-expression genes and links in the dysfunctional pathways and other HCV-related biological processes.

Figures 3b, 3d and 4b shows another application of *C*-score in the dataset of H3N2 virus-induced influenza infection, in which there are 16 sampling time points over the whole study period (132 hours). Nine subjects were diagnosed as having influenza infection or corresponding clinic symptoms 45 hours after the exposure to influenza viruses [26]. The specific procedure of data processing, gene filtering and computation are similar to the previous application. It can be seen that the *C*-score curves based on the human PPI network for live influenza infection in Fig. 3b, with eight probability curves respectively corresponding to the first eight candidate points. Among the probability curves in Fig. 3b, the red curve presents the *C*-score based on the top 5 % differential-expression genes at 29 hr, while the orange one shows that calculated at 36 hr, the adjacent time point after 29 hr. Both these two curves show a sudden decrease of consistence probability during the progression, which implies that the onset of pre-disease state is around a period spanned from 29 to 36 hr, i.e., the upcoming deterioration into a disease state might be after 36 hr, which is in coincidence with the fact that the early symptoms of influenza infection arises after 45 hr. Furthermore, to show the significance of the selected genes whose collective dynamics results in the significant changes in the links and thus generate the earliest signal at 29 hr, in Fig. 3d we carried out the clustering analysis based on the correlation values at 29 hr. We see that the genes in the selected group show a large fold change and a significant *P*-value in average. For these genes, the enrichment analysis shows that the most significant pathway is influenza A pathway, which shows the involvement of the selected genes in the biological processes of infection.

Figure 4b presents the dynamical evolution of the gene network respectively at 5, 12, 29, 60 hr for live influenza infection. The selected gene group in Fig. 3d is placed in the top corner. It can been seen that at 29 hr the structure of the subnetwork of the selected genes changes significantly and thus signals the upcoming deterioration into a disease state which is also in coincidence with the clinic observation.

Therefore, our application results are in coincidence with the experimental observation and successfully detect the early-warning signal of the impending critical transition.

## Discussion and conclusions

Complex diseases significantly damage the health of people all over the world. Detecting the early-warning signal of the sudden deterioration provides an opportunity to interrupt and prevent the continuing costly cycle of managing these diseases and their complications. Although it is crucial to detect the pre-disease state so as to prevent the qualitative deterioration by taking appropriate intervention actions, it is a challenging task to reliably identify the pre-disease state because the state of the system may show neither apparent change nor clear phenomenon before this critical transition during the disease progression. This is also the reason why diagnosis based on traditional biomarkers may fail to indicate a pre-disease state.

In this work, by detecting the dynamical change of links in a network, we presented a computational method and corresponding algorithm based on HMM to measure the dynamical difference of the system progression, and thus identify the imminent critical transition. It is worth noting that this method aims to detect the early-warning signal generating from the pre-disease state (or pre-transition state), rather than to find the indication of disease state (or after-transition state) in which the qualitative deterioration has already taken place.

We applied our method to the identification of the pre-disease state based on a simulated dataset and two microarray datasets, which demonstrate the sensitivity and effectiveness of our method. For both two diseases, we constructed bio-molecular networks (Fig. 4) to gauge the dynamical regulation among genes at different sampling point along a time-course progression. Both the functional and enrichment analyses validate the computational results. Therefore, the HMM-based method provides a computational possibility of prying into the underlying mechanism of biological processes of the disease progression, and thus may help to achieve the timely intervention. Our dynamic network analysis also suggests, in regard to the diseases, to focus on the specific pre-disease states to probe the in situ external perturbation (such as environment changes) preceding the development into a badly ill stage. This may lead to not only insights of external environment interactions, but also an effective time window for novel intervention or therapeutic strategies in specific diseases. The main difference between our work and previous ones is that rather than screen out some variables (genes or proteins), the proposed method mainly focuses on the direct identification of critical transition point, by calculating and comparing the consistence probability of each candidate end point of the Markov model in the normal state. Therefore, the accuracy of HMM-based approach is not limited by the selection of features. This is the main value in the potential applications of the HMM-based method from a network point of view.

There are limitations of this work. First, the validity of the identified pre-disease state and the accurate result needs further supports from animal experiments or clinical studies. Second, the method is insensitive when the correlations are not differentially expressed. Although this work is merely a step towards detecting the early-warning signals of critical transition during disease progression and the algorithm is expected to be improved in both sensitive and accurate ways, it opens a window of opportunity for the applicable approach to the early-warning system of critical transition during the biological processes of complex diseases.
